# Performance of Newly Developed Weed-Competitive Rice Cultivars under Lowland and Upland Weedy Conditions

**DOI:** 10.1017/wsc.2017.57

**Published:** 2017-11-01

**Authors:** Niña Gracel B. Dimaano, Jauhar Ali, Pompe C. Sta. Cruz, Aurora M. Baltazar, Maria Genaleen Q. Diaz, Bart L. Acero, Zhikang Li

**Affiliations:** First, third, fourth, and fifth authors: Graduate Student, Professor 6, Adjunct Professor, and Professor 3, University of the Philippines-Los Banos, Los Banos, Laguna 4031, Philippines; second and sixth authors: Senior Scientist and Researcher, International Rice Research Institute, Los Banos, Laguna 4031, Philippines; seventh author: Chief Scientist, Chinese Academy of Agricultural Sciences, Beijing, China

**Keywords:** G-6 introgression lines, breeding, weed-competitive ability

## Abstract

Four early-generation backcross populations (BC1F2) derived from one common recipient parental background, Weed Tolerant Rice 1 (‘WTR1’), and four different donor parents (‘Y134’, ‘Zhong 143’, ‘Khazar’, and ‘Cheng Hui-448’) were tested to identify suitable donor and recipient parents for weed competitiveness and to standardize evaluation of the weed-competitive ability in rice. ‘GSR IR2-6’ (G-6) derived from a backcross of WTR1/Y134//WTR1 was selected as the best population and was advanced for phenotypic experiments in the 2014 dry season. The introgression lines (ILs) derived from the G-6 population were evaluated for seed germination and seedling vigor in greenhouse conditions and for weed-competitive ability under field conditions (upland weed-free, upland weedy, and lowland weedy). Parents and checks were included for comparison. Selection pressure for weed competitiveness was relatively stronger in upland conditions than in lowland conditions. After three rounds of selection and based on their relative grain yield performances across conditions, a total of 21 most-promising introgression fixed lines showing superior traits and weed-competitive ability were identified. G-6-L2-WL-3, G-6-RF6-WL-3, G-6-L15-WU-1,G-6-Y16-WL-2, and G-6-L6-WU-3 were the top ILs in lowland weedy conditions, whereas G-6-Y7-WL-3, G-6-Y6-WU-3, G-6-Y3-WL-3, and G-6-Y8-WU-1 were the highest yielding in upland weedy conditions. The use of weed-competitive rice cultivars in African and Asian countries will be a highly effective strategy to reduce production costs and provide alternative solutions to the unavailability of herbicides. Competitive rice varieties will also significantly improve grain yields in aerobic rice systems and can become an important strategy for successful upland rice production.

**Nomenclature:** Rice, *Oryza sativa* L.

Rice is the main staple food and source of income for most people in the world, particularly in Asia and Africa. In 2014, the world’s rice production was estimated at 750.9 million tons, of which 678.8 million tons and 28.3 million tons were harvested in Asia and Africa, respectively (Food and Agriculture Organization 2014).

Rice is generally cultivated in irrigated conditions for ease of weed control and better establishment. Declining water availability combined with erratic climatic conditions (El Niño) is becoming a major concern that threatens the sustainability of irrigated rice ecosystems (Rahman et al. 2012). Thus, the aerobic rice system and water-saving techniques are being developed and promoted to mitigate the problem of water scarcity (Belder et al. 2005; Bouman 2001). However, weed problems present a major constraint that contributes to the greatest yield loss in aerobic rice systems (Chauhan et al. 2015; Jabran and Chauhan 2015; West Africa Rice Development Association 1996). Aerobic soil conditions are highly conducive to weed seed germination and growth, which result in greater weed pressure and higher yield losses than in flood-irrigated rice (Balasubramanian and Hill 2002; Rao et al. 2007). Failure to control weeds in aerobic rice often results in very low or zero yield (Johnson 2009). In sub-Saharan Africa, weeds lead to an estimated rice yield loss of 2.2 million tons per year (Rodenburg and Johnson 2009), whereas in the tropics, yield losses are estimated to be at 35% (Oerke and Dehne 2004).

The most common control measure against weeds is hand weeding, which is laborious and expensive. Hand-weeding operations (two to three times per cropping season) comprise 15% of the total farming operation cost (VanDevender et al. 1997). Nowadays, the use of herbicides to control weeds is considered to be the most practical, effective, and economical approach (Rahman et al. 2012). However, the intensive use of herbicides has led to negative results, such as herbicide resistance, environmental contamination, and weed shifts toward tolerant ecotypes (Carey et al. 1995; Fisher et al. 1993; Lemerle et al. 2001; Valverde et al. 2000).

Breeding for weed-competitive rice cultivars (with high yield ability) can be an important strategy for reducing hand weeding and herbicide inputs (Zhao et al. 2006a). However, limited studies have been carried out to evaluate weed competitiveness in various crops, including corn (Zea mays L.) (Silva et al. 2010), sorghum [Sorghum bicolor (L.) Moench] (Wu et al. 2010), canola (Brassica napus L.) (Lemerle et al. 2010), soybean [Glycine max (L.) Merr.] (Jannink et al. 2000; Place et al. 2011), wheat (Triticum aestivum L.) (Huel and Hucl 1996; Lanning et al. 1997; Lemerle et al. 1996, 2001), and rice (Moukoumbi et al. 2011; Namuco et al. 2009; Zhao et al. 2006a, 2006b).

Weed-competitive ability is a complex trait that cannot be attributed to a single characteristic but is a result of the interaction among several desirable traits (Caton et al. 2000; Chauhan et al. 2015; Kruepl et al. 2007), thus making it difficult for plant breeders to breed for weed-competitive crop cultivars. Consequently, it is imperative to determine the complexity of the mechanisms and agro-morphological traits that confer weed competitiveness to fast-forward breeding efforts for weed-competitive rice cultivars. This can be done by identifying suitable germplasm for weed competitiveness and standardizing the screening protocol for phenotyping traits related to weed competitiveness.

This study was conducted to identify suitable donors and recurrent parents for weed competitiveness, identify and standardize the traits related to weed competitiveness, and identify promising introgression lines (ILs) with superior and competitive traits.

## Materials and Methods

**Place and Date of Study.** Laboratory, greenhouse (NG-04), and field experiments (lowland/irrigated: 14.23°N, 121.43°E; upland/dry seeded: 14.22°N, 121.43°E and 23 m elevation) were conducted at the International Rice Research Institute (IRRI) Los Baños, Laguna, Philippines, during the 2012 dry season (DS), 2013 wet season (WS), and 2014 DS. The soil type on the experimental farm is Maahas clay loam (isohyperthermic mixed Typic Tropudalf).

**Selection of Plant Materials.** Four early-generation, backcross populations (BC1F2) derived from one common recipient parental varietal background of Weed Tolerant Rice 1 (WTR1) and four different donor parents (Y134, Zhong 143, Khazar, and Cheng Hui-448) were tested to identify a suitable donor parent and recipient parent for weed competitiveness. The germplasm lines were selected based on grain yield performance and selection criteria for weed competitiveness (Table 1). The cultivars used as a control in the investigation of weed-competitive ability traits are presented in Table 2.

**Table 1:** Criteria in the selection of Green Super Rice (GSR) populations used in the investigation of weed competitive ability (WCA) traits in rice.^a^

Criteria	Theoretical WCA ability	Candidate population
1.Higher BC1F2 bulk population grain weights under RF and Y conditions.	Desirable response to indirect selection for WCA while having desirable yielding potential.	G-6 Donor parent: Y134 No. of lines: 48 16(Y), 15(L), 17(RF)!
2. Higher BC1F2 bulk population grain weights under Y and L conditions.	Essential in studying the WCA trait.	G-7 Donor parent: Zhong413 No. of lines: 43 14(Y), 15(L), 14(RF)
3. Higher BC1F2 bulk population grain weights under RF condition only.	Desirable response to indirect selection for WCA trait.	G-8 Donor parent: Khazar No. of lines: 47 16(Y), 15(L), 16(RF)
4.igher BC1F2 bulk population grain weights under Y condition only.	No desirable response to indirect selection for WCA trait.	G-4 Donor parent: Cheng Hui-448 No. of lines: 46 16(Y), 14(L), 16(RF) Total no. of lines: 184

a Conditions: L, low-input; RF, rainfed conditions; Y, irrigated.

**Table 2:** List of the check cultivars used in the investigation of weed competitive ability (WCA) traits in rice.

No.	Cultivar name	Description^a^
1	Weed Tolerant Rice 1	GSR recurrent parent
2	Cheng Hui-448 (G-4), Y134 (G-6), Zhong413 (G-7), and Khazar (G-8)	Donor parents
3	PSB Rc82	NCT transplanted and direct-seeded rice check
4	NSIC Rc222	IRRI irrigated check
5	GSR IR1-8-S6-S3-Y2	IRRI GSR variety
6	NSIC Rc192	NCT rainfed/upland check
7	IR74371-70-1-1	IRRI drought-tolerant check
8	Apo	IRRI upland and aerobic rice cultivar

a Abbreviations: GSR, Green Super Rice; IRRI, International Rice Research Institute; NCT, National Rice Cooperative Tests.

**Figure 1:**
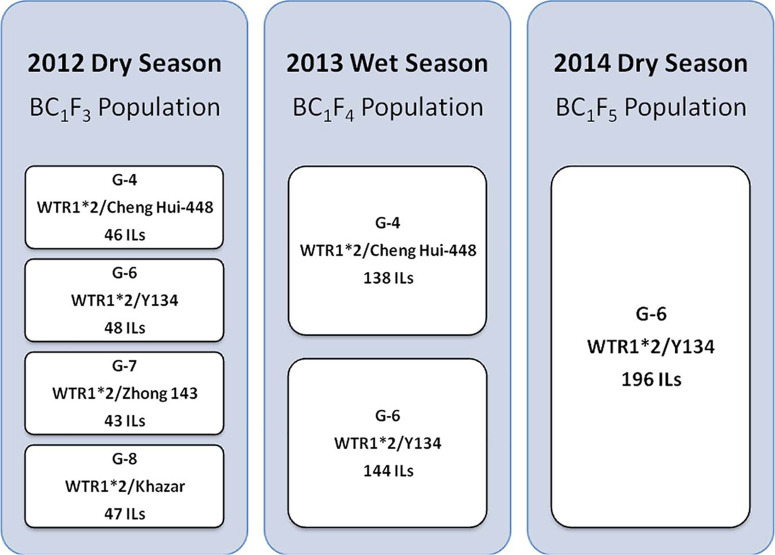
Green Super Rice (GSR) populations used in the weed-competitive ability experiment.

**Selection Scheme.** In the 2011 WS (before this study), four early-generation backcross populations (BC1F2) derived from one common recipient background (WTR1) and four different donor parents (Y134, Zhong 143, Khazar, and Cheng Hui-448) were initially tested to identify suitable donor and recipient parents for weed competitiveness. These BC1F2 populations were designated by a Green Super Rice (GSR) name as follows: G-4 (WTR1/Cheng Hui-448//WTR1), G-6 (WTR1/Y134//WTR1), G-7 (WTR1/Zhong 413//WTR1), and G-8 (WTR1/Khazar//WTR1). These BC1F2 populations were evaluated and selected under irrigated (Y), rainfed (RF), and low-input (L) conditions to produce the BC1F3 generation. Promising ILs were selected from each population by single plant selection. The G-4 population has 46 promising ILs: 16 (Y), 14 (L), and 16 (RF); G-6 has 48 ILs: 16 (Y), 15 (L), and 17 (RF); G-7 has 43 ILs: 14 (Y), 15 (L), and 14 (RF); and G-8 has 47 ILs: 16 (Y), 15 (L), and 16 (RF). In total, 184 BC1F3 ILs were used for the 2012 DS selection process under three conditions: upland weed-free (UPWF), upland weedy (UPW), and lowland weedy (LLW). In the 2013 WS, the BC1F4 populations were reduced to G-4 (138 ILs) and G-6 (144 ILs) (Figure 1). Ultimately, the G-6 population was advanced to the 2014 DS and was used to standardize the evaluation criteria for weed-competitive traits in rice.

**Figure 2:**
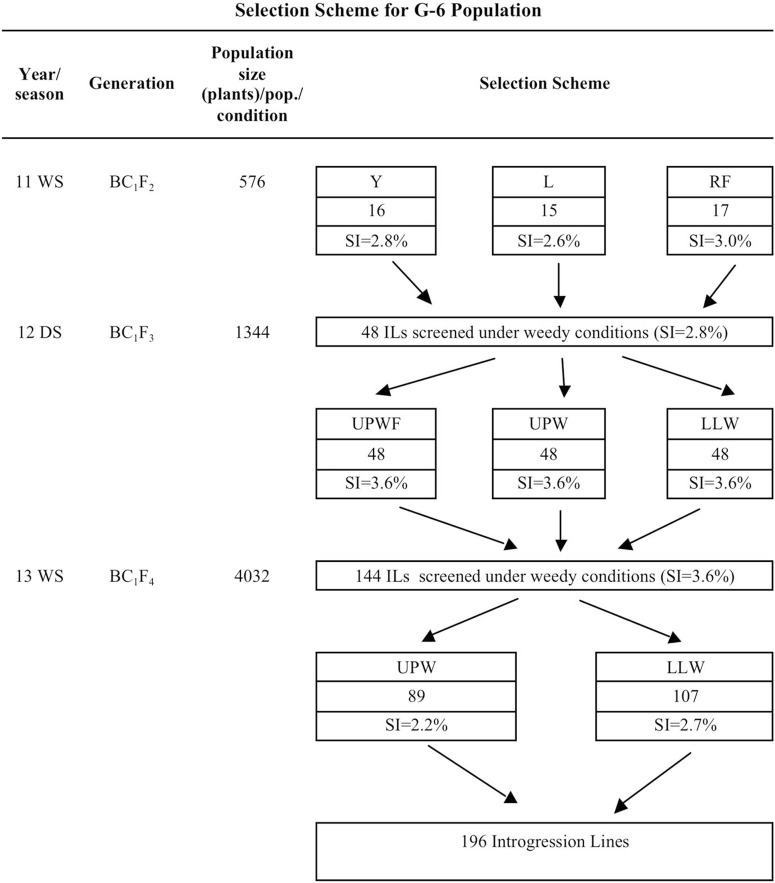
Selection scheme for the GSR IR2-6 (G-6) population used in the weed-competitive experiment. DS, dry season; ILs, introgression lines; L, low input; LLW, lowland weedy; Pop., population; RF, rainfed; SI, selection intensity; UPWF, upland weed-free; UPW, upland weedy; WS, wet season; Y, irrigated.

Selection intensity (SI) is the number of single plants selected from a given population size of segregating plants planted in a condition and is expressed as a percent. G-6 BC1F2 population size in the 2011 WS was 576 to create an SI of 2.8% (Y), 2.6% (L), and 3.0% (RF) (Figure 2). In the 2012 DS, SI was 3.6% from a population size of 1,344 across all conditions, whereas in the 2013 WS, SI was 2.2% in UPW (89 ILs) and 2.7% in LLW (107 ILs) (Figure 2). In the 2014 DS, 196 ILs from the G-6 BC1F5 population were planted in UPW, UPWF, and LLW and two batches were grown under greenhouse conditions to determine their weed-competitive ability and identify promising ILs to be developed as competitive rice varieties.

**Experimental Design and Treatments.** BC1F3 to BC1F5 populations from four different donor parents derived in one common WTR1 recipient background (subplots) were tested for weed competitiveness under main plots: UPWF, UPW, and LLW conditions. The experimental design is an augmented randomized complete block design (RCB) with two replications (due to the limited number of seeds). A greenhouse experiment, seed germination test, and seedling vigor test (replicated twice in an RCBD) were also conducted in the 2014 DS to strengthen selection for weed-competitive traits.

**Field Experiments.** The G populations with a seeding rate of 90 viable seeds were manually drilled in four rows (7 seeds row–1 in 20-cm row spacing by 15-cm plant-to-plant spacing). The UPWF conditions were treated with the PRE herbicide oxadiazon (Ronstar EC25, 0.75 kg ai ha–1, Bayer CropScience, Canlubang Industrial Estate, Laguna, Philippines) using a backpack sprayer (TeeJet® 200015, TeeJet Technologies, Wheaton, IL) mounted with a flat-fan nozzle calibrated to deliver 160 L ha–1 at 138 kPa. Herbicide applications were made just after seeding. Plots were irrigated using a sprinkler immediately after herbicide application and were maintained weed-free during the critical period of competition. The LLW and UPW conditions were completely hand weeded only once at 21 d after sowing (DAS). Weeds were allowed to grow thereafter. To augment and homogenize the natural weed population in the LLW trial, barnyardgrass [Echinochloa crus-galli (L.) Beauv.] seeds were broadcast at a rate of 15 kg ha–1, whereas in the upland weedy trial, itchgrass [Rottboellia cochinchinensis (Lour.) W. D. Clayton] seeds were broadcast at a rate of 10 kg ha–1 in the 2012 DS field experiment. Weed seeds were broadcast at 28 d after rice was sown.

In each field, a compound N–P–K fertilizer (14:14:14) was applied at 200 kg ha–1, and two additional applications of urea were topdressed each at 60 kg ha–1 at 28 and 56 DAS, respectively. The UPWF and UPW fields were irrigated regularly with an overhead sprinkler and were maintained under nonsaturated aerobic conditions to facilitate the growth of weeds.

Rice agro-morphological traits, yield, and weed-related component traits were evaluated for weed competitiveness under field conditions. For plant height and tiller number, five random plants were measured for each IL. For yield traits, three random plants from each IL were selected and measured for panicle number, flag-leaf length, and flag-leaf width. Also, the three selected plants were threshed and separated into filled and unfilled grains, which were then counted and weighed.

The predominant weed species under weedy conditions (UPW and LLW) were determined by computing the summed dominance ratio (SDR) of each species based on weed density, relative density, biomass, relative biomass, and frequency per square meter. Three samples (1-m2 quadrats) were taken in UPW and two in LLW conditions.

The most dominant weed species in UPW conditions based on SDR values were purple nutsedge (Cyperus rotundus L.) (53.06%), goosegrass [Eleusine indica (L.) Gaertn.] (44.63%), guineagrass [Urochloa maxima (Jacq.) R. Webster] (37.97%), eclipta [Eclipta prostrata (L.) L.] (37.47%), and knotgrass (Paspalum distichum L.) (26.97%). The most dominant weed species in LLW conditions based on SDR were fimbry (Fimbristylis littoralis Gaudich.) (83.89%), junglerice [Echinochloa colona (L.) Link] (43.14%), Chinese sprangletop [Leptochloa chinensis (L.) Nees] (34.96%), eclipta (34.22%), and guineagrass (34.01%). The externally seeded weed species barnyardgrass had a 17.8% SDR value, but no data were collected for itchgrass, because random sampling (sample size of 3) was done across the experimental plot.

**Greenhouse Experiments.** Two greenhouse experiments were conducted to determine the traits related to weed competitiveness in the G-6 population. In the first experiment, two replications of 5 seeds of G-6 ILs (BC1F5 population), parents WTR1 and Y134, and checks PSB Rc82, NSIC Rc222, NSIC Rc192, GSR IR1-8-S6-S3-Y2, IR74371-70-1-1, and Apo were direct seeded in plastic pots filled with soil, thinned to 1 seedling pot–1, and maintained until grain maturity. One hundred seeds of junglerice were sown in each pot simultaneously with rice seeds to homogenize weed emergence. The initial germination rate of each IL, parents, and checks was measured at 7 DAS. Plant height of rice seedlings was recorded at 7, 14, 21, and 28 DAS. At maturity, data pertaining to maximum plant height, tiller number, panicle fresh weight, flag-leaf length, flag-leaf width, leaf area, number of panicles, number of filled and unfilled grains, spikelet fertility, and grain weight of each IL, parents, and checks were collected.

In the second experiment, two replications of 5 seeds of G-6 ILs (BC1F5 population), parents WTR1 and Y134, and checks were direct seeded and maintained in plastic pots filled with field soil for 28 DAS to investigate traits related to seedling vigor. One hundred seeds of junglerice were sown in each pot simultaneously with rice seeds to homogenize weed emergence. The initial number of germinated seeds and percent germination of each IL, parents, and checks were gathered. Rice seedling height and number of leaves were measured at 7, 14, 21, and 28 DAS. At 28 DAS, the number of tillers and leaf chlorophyll content (Soil-Plant Analyses Development [SPAD] meter readings) were recorded. After each observation, the rice seedlings were pulled out carefully for measurement of shoot and root length. The lengths of shoots and roots were measured from the collar region down to the tip of the longest root and shoot of each seedling. The leaves and roots of each seedling were separated for leaf fresh and dry weights, root fresh and dry weights, and total fresh and dry weights. A vigor index was computed by multiplying percent germination by seedling total dry weight (Diwan 2006). Weed density, fresh weight, and dry weight data were also collected to correlate with seedling vigor performance as related to weed competitiveness. Weed biomass was clipped at the soil surface from each pot, weighed, and oven-dried at 70 C for 5 d to obtain dry weight data.

**Seed Germination Tests.** Seed dormancy was broken by subjecting the seeds to 50 C temperature for 4d (Jennings and de Jesus 1964). Two replications of 25 seeds were germinated in a 9-cm-diameter petri dish lined with a filter paper and placed in a germination chamber for 14 d. Seeds showing 2-mm radicle length were considered germinated. Seeds that germinated after 48 h were counted and recorded as first-count germination. Seeds that germinated after 7 d were counted and recorded as second-count germination. The rate of germination was calculated as the ratio of the final count of germination to the total number of initial seeds and expressed as a percentage. On the day 14, leaf and root lengths were recorded from five normal germinated seeds randomly selected from each replication. Leaf length was measured from the collar region to the tip of the longest leaf and expressed in centimeters. Root length was measured from the collar region down to the tip of the longest root. Average fresh and dry weights were derived from the total fresh and dry weights of germinated seeds (in milligrams) divided by the number of germinated seeds. Vigor index was calculated by multiplying the rate of germination by total dry weight.

**Statistical Analysis.** Statistical analysis was performed using R v. 1.5 (R Core Team 2016) and PBTools 1.4 (IRRI 2017). Descriptive statistics, correlation analysis, and ANOVA were performed on all data collected (Table 3). Pearson’s correlation test was carried out to assess possible variable linear relationships across weedy conditions. Analysis of variance was performed to verify whether the weed-competitive ability traits of at least one of the ILs varies with weed regimes. The ILs were analyzed for the significance of genotypic effect per weedy condition. Pairwise mean comparison using Fisher’s least significant different test was used to analyze the yield differences among the top-performing ILs, parents, and check cultivars. Regression analysis was carried out to determine which traits were significantly affecting the grain yield of the newly developed ILs tested under weedy conditions. Using R v. 1.5, the regression model was selected using all possible regression models with leaps and the coefficient of variation (R^2^) as the criterion.

**Table 3:** Rice agro-morphological characters, vigor indexes, yield-related traits, and weed components evaluated for weed-competitive ability experiment.

No.	Rice component	Description
1	First-count germination	No. of germinated seeds after 48 h
2	Second-count germination	No. of germinated seeds after 7 d
3	Percent germination	Ratio of the first count to the final count of germination
4	Shoot length (cm)	Measured from the collar region to the tip of topmost leaf
5	Root length (cm)	Measured from collar region down to the tip of the longest root
6	Total FW of germinated seeds	Total fresh weight of all seeds that germinated
7	Total DW of germinated seeds	Total dry weight of all seeds that germinated
8	Average fresh weight	Fresh weight/no. of seeds that germinated
9	Average dry weight	Dry weight/no. of seeds that germinated
10	Leaf fresh weight (g)	Fresh weight of leaves
11	Root fresh weight (g)	Fresh weight of roots
12	Total fresh weight (g)	Measured by computing leaf fresh weight + root fresh weight
13	Leaf dry weight (g)	Dry weight of leaves after drying at 65 C for 3 d
14	Root dry weight (g)	Dry weight of roots after drying at 65 C for 3 d
15	Total dry weight (g)	Measured by computing leaf dry weight + root dry weight
16	Vigor Index	Measured by computing percent germination × seedling dry weight
17	Seedling plant height (cm)	Plant height at 7, 14, 21, and 28 days after sowing
18	Seedling no. of leaves	No. of leaves at 7, 14, 21 and 28 days after sowing
19	Max. plant height (cm)	Measured as the distance from the ground to the panicle tip
20	No. of tillers	No. of tillers
21	Leaf chlorophyll content (LCC)	Leaf chlorophyll content based on Soil-Plant Analyses Development (SPAD) meter
22	No. of panicles	No. of panicles
23	Flag-leaf length (FLL) (cm)	Measured from the base to the tip of flag leaf
24	Flag-leaf width (FLW) (cm)	Measured from the mid-section of flag leaf
25	Leaf area (cm2)	Measured by computing 0.75 × FLL × FLW
26	No. of filled grain	Grains that are undamaged and filled
27	No. of unfilled grain	Grains that are damaged and unfilled
28	Panicle fresh weight (g)	Fresh weight of panicles
29	Single plant yield (g)	Harvested, dried, weighed, and adjusted to 14% moisture content
30	% Spikelet fertility	Measured by computing filled × 100/filled + unfilled grains
31	Weed tolerance index	Trait under weedy condition – trait under weed-free condition/trait under weedy condition
	Weed component	Description
32	Weed density	No. of weeds
33	Weed biomass	Fresh weight and dry weight
34	Weed frequency	Number of weed species observed/number of samples taken
35	Summed dominance ratio	Relative density + relative fresh weight + weed frequency/3

## Results and Discussion

**Novel Weed-Competitive Rice-Breeding Strategy.** Breeding for weed-competitive rice is becoming an important area of interest to plant breeders and weed scientists because of the increasing weed problems in aerobic rice ecosystems, adverse effects of extensive herbicide use, increasing reports of herbicide resistance, herbicide contamination of the environment, lack of labor for hand weeding, and inaccessibility to herbicides in marginal areas. To overcome this, we need to breed weed-competitive cultivars that could suppress or tolerate weeds without compromising grain yield. Weed-competitive cultivars will complement other weed control methods by suppressing weeds while maintaining acceptable yields (Worthington and Reberg-Horton 2013). Successful breeding of weed-competitive rice cultivars will significantly reduce rice production costs in aerobic rice systems through a reduction in herbicide use and hand weeding. This strategy could further augment water-saving techniques to reduce production costs and improve yields in upland rice production.

Breeding programs for cultivars that possess weed-competitive ability will be advanced by satisfactory evaluation and germplasm screening for weed competitiveness (Kruepl et al. 2007). To achieve success in germplasm screening, evaluation criteria should be measured in a nondestructive and rapid process (Caton et al. 2003). Moreover, the key traits that confer weed competitiveness need to be correctly identified and elucidated (Gill and Coleman 1999).

Selection for weed-competitive ability can be implemented directly (under weedy conditions) or indirectly (under weed-free conditions) for secondary traits related to weed competitiveness (Zhao et al. 2006a). Traits under weedy and weed-free conditions can be considered as correlated traits, expressed by a single genotype in two diverse environments (Zhao 2006). All traits gathered under weed-free conditions were closely correlated with the same traits gathered under weedy conditions (Zhao et al. 2006b). Moreover, traits that are considered useful in indirect selection for weed competitiveness should be highly correlated with yield in weedy conditions and practical for use in large breeding populations to attain adequate SI (Atlin et al. 2001). Indirect selection for weed competitiveness under weed-free conditions permits germplasm selection to be implemented at the beginning of the breeding program, while direct selection under weedy conditions can be employed only in the later phase, when a satisfactory number of seeds are available (Wall 1983). Based on these criteria, the candidate populations in this experiment were selected through direct (under UPW and LLW) and indirect (under UPWF) selection procedures with satisfactory SI. The weed-competitive traits in rice were further confirmed and standardized in a controlled greenhouse experiment for seedling vigor tests and analyzed for correlation with weed density, weed fresh weight, and weed dry weight.

Severe SI is extremely useful for identifying and selecting elite ILs only in each stage and in different conditions as compared with the performance of both the parents and check varieties. In our breeding program on weed competitiveness, we ensured a stronger SI of less than 3.6% under all conditions and stages. SI of 3.6% is the percentage of the number of single plants selected (48) from a given population size of segregating plants (1,344) planted in each weedy condition. Each elite selection for weed competitiveness was based on seedling vigor, rapid growth, effective tiller number, and singleplant grain yield. The parent Y134 (donor) and WTR1 (recipient) were identified as the most suitable parents for weed competitiveness. A single backcross population (G-6) from a cross between Y134 and WTR1 was identified as the best population, as it expressed both weed competitiveness and high yield potential under weedy conditions in lowland and upland environments.

Among the selections, G-6-L2-WL-3, G-6-RF6-WL-3,G-6-L15-WU-1, G-6-Y16-WL-2, and G-6-L6-WU-3 were the top ILs in LLW conditions, whereas G-6-Y7-WL-3, G-6-Y6-WU-3, G-6-Y3-WL-3, and G-6-Y8-WU-1 were the highest-yielding ILs under upland weedy conditions.

**Selection Pressure by Environment.** Initial screening under Y, RF, and L conditions allowed the selection of 48 promising G-6 ILs that gave relatively higher BC1F2 bulk population grain weights under both rainfed and irrigated environments. This correlates to their desirable response to indirect selection for weed competitiveness while having desirable yield potential. G-7 ILs gave higher BC1F2 bulk population grain weights under irrigated low-input environments, while G-8 ILs had higher BC1F2 bulk population grain weights under rainfed conditions only. However, the ILs of G-7 and G-8 did not perform well when tested under UPWF, UPW, and LLW conditions in the 2012 DS. Thus, these two populations were dropped in the 2013 WS. G-4 ILs gave higher BC1F2 bulk population grain weights under irrigated conditions only, signifying that these ILs have no desirable response to indirect selection for weed-competitive ability. When tested under UPWF, UPW, and LLW conditions in the 2012 DS, both G-4 and G-6 performed well; thus, selected ILs from these populations were advanced for further screening in the 2013 WS. Finally, G-6 ILs were selected as the best population demonstrating weed-competitive ability under LLW and UPW conditions and expressing higher grain yield potential than both the parents and inbred checks.

**Figure 3:**
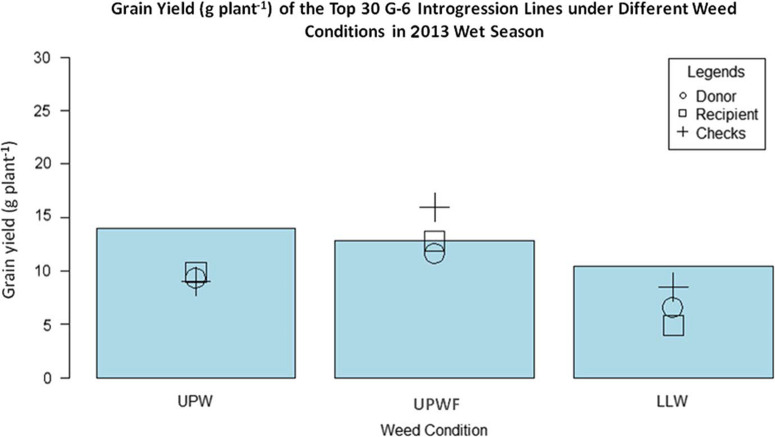
Grain yield (g plant–1) of top 30 GSR IR2-6 (G-6) introgression lines (ILs), recipient parent, donor parent, and checks under upland weedy (UPW), upland weed-free (UPWF), and lowland weedy (LLW) conditions in the 2013 wet season. Bars represent mean grain yield (g plant–1) of the G-6 ILs.

*Performance of G-6 ILs in UPWF, UPW, and LLW Conditions in the 2013 WS*.Figure 3 shows the grain yield of the top 30 G-6 ILs, WTR1 (recipient parent), Y134 (donor parent), and checks PSB Rc82 and Apo in the 2013 WS. In UPW conditions, the ILs had the highest grain yield, but there was no significant difference in the grain yields of WTR1, Y134, and the checks, which all performed significantly below the ILs. The ILs from the G-6 populations were better weed competitors than both the parents and the checks. Thorough study of the different traits of these identified ILs will help elucidate the key traits for weed competitiveness in rice.

In UPWF conditions, the checks surpassed the grain yields of both the parents and ILs. This is expected, since the checks were adapted to weed-free environments. However, with the presence of weeds in upland conditions, the yield of the checks and both WTR1 and Y134 decreased significantly, with greater yield reductions in the checks compared with WTR1 or Y134. The checks were sensitive to weeds; therefore, their yields declined drastically under weedy conditions.

In LLW conditions, the grain yield of the ILs was greater than that of both the parents and checks, although significantly lower than the grain yields obtained under upland conditions. The checks still outperformed both parents (WTR1 and Y134), while the recipient parent (WTR1) had the lowest grain yield.

There was no significant difference in the grain yields of ILs subjected to UPWF and UPW conditions in the 2013 WS (Table 4). The ILs had strong weed-competitive ability and were able to produce comparable yields even with the presence of weeds. This observation was further evaluated in field and greenhouse experiments in the 2014 DS.

**Table 4:** Grain yield comparison of top 30 GSR IR2-6 introgression lines from upland weedy, upland weed-free, and lowland weedy conditions in 2013 wet season (WS) and 2014 dry season (DS).

Treatment	Grain yield^a^	
	2013 WS	2014 DS
	**————g plant**^–1^**————**	
Upland weed-free	12.86 ab	7.06 a
Upland weedy	13.97 a	4.25 b
Lowland weedy	10.43 b	0.77 c

a Weedy conditions with the same letters are not significant at the 5% level.

**Figure 4:**
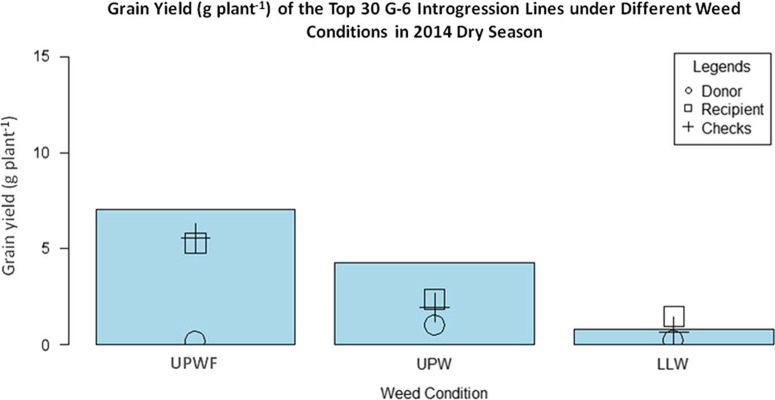
Grain yield (g plant–1) of top 30 GSR IR2-6 (G-6) introgression lines (ILs) under upland weedy (UPW), upland weed-free (UPWF), and lowland weedy (LLW) conditions in the 2014 dry season. Bars represent mean grain yield (g plant–1) of the G-6 ILs.

*Performance of G-6 ILs in UPWF, UPW, and LLW Conditions in the 2014 DS*.Figure 4 shows the grain yield of the top 30 G-6 ILs, WTR1 (recipient parent), Y134 (donor parent), and checks PSB Rc82 and Apo in the 2014 DS. In UPWF conditions, the ILs had the highest grain yield, whereas Y134 produced only 0.2 g grain yield plant–1 due to bacterial blight disease. The grain yields of the recurrent parent WTR1 and the checks showed nonsignificant differences.

In UPW conditions, the grain yields of the ILs were significantly lower in the presence of weeds. As expected, the selected ILs had the highest grain yield, whereas Y134 had the lowest yield. The grain yields of WTR1 and the checks did not differ and were significantly lower than those of the ILs. In LLW conditions, the grain yield of the ILs declined significantly. Recurrent parent WTR 1 had the highest grain yield, whereas Y134 had the lowest. However, the grain yield of Y134 did not differ from that of the check cultivars. There was a significant difference in the grain yields of ILs subjected to UPWF, UPW, and LLW conditions (Table 4). The highest grain yields were observed in UPWF conditions. However, even with the presence of weeds under UPW conditions, the ILs produced high yields, suggesting that the selected ILs have a strong weed-competitive ability. Consequently, even with less hand weeding or herbicide application, the yields of the ILs will just be the same or higher. In the long run, this is efficient in terms of lower labor cost, less herbicide use, and more profit. However, the ILs were not performing well in LLW conditions. The selected ILs seemed to be more adapted to upland conditions, and the selection for weed-competitive cultivars is stronger for UPW conditions. The first few cycles of selection under LLW conditions and using parental lines that perform well under LLW conditions may be important. These UPW and LLW conditions are two extreme conditions, and selection needs to be done separately in a more systematic manner involving crosses with ideally suitable parental lines.

The different agronomic and competitive traits of these ILs from the 2014 DS field experiment were further investigated under controlled environments including a greenhouse experiment, a seedling vigor test, and a germination test.

**Key Traits for Weed-Competitive Ability**

*Correlation Analysis for Weed-Competitive Ability Traits under Field Conditions in the 2013 WS*. High yield capacity despite the presence of weeds is a strong indication of weed-competitive ability in cultivars. In the 2013 WS, grain yield was regarded as the key factor in considering selection for weed competitiveness. Correlation analysis showed that tiller number (P < 0.001; r = 0.49) and panicle number (P < 0.001; r = 0.64) are significantly and positively correlated with grain yield (Table 5). This indicates that ILs with more tillers and panicles tend to have higher yield. In contrast, plant height (r = –0.02), leaf chlorophyll content (r = –0.06), or panicle length (r = –0.13) did not show a significant correlation (P > 0.05) with grain yield. Plant height is a key trait for weed competitiveness (Lanning et al. 1997; Lemerle et al. 1996). However, the result for the 2013 WS showed that taller plants will not necessarily give higher grain yield. Fischer et al. (1997, 2001) reported that there is no clear association between plant height and weed-suppressive ability in irrigated and upland rice. Similarly, having higher leaf chlorophyll content (SPAD reading) and longer panicles is not associated with grain yield. Moukoumbi et al. (2011) had observed weed-competitive cultivars showing a SPAD unit of less than 30, whereas in this study, results of the 2013 WS experiment showed a SPAD mean unit of 37.12. Negative correlation exists between panicle number and panicle length, but tiller number is positively correlated with panicle number (P < 0.001; r = 0.55) and negatively correlated with panicle length (P < 0.05; r = –0.26). Conversely, leaf chlorophyll content (SPAD reading) is positively correlated with panicle length (P < 0.05; r = 0.23).

**Table 5:** Correlation analysis of traits of the top GSR IR2-6 introgression lines measured during the 2013 wet-season field experiment.^a^

	Grain yield	Plant height	Tiller no.	Leaf chlorophyll content	Panicle no.
	—g plant–1—	—cm—			
				
Grain yield (g plant–1)					
Plant height (cm)	–0.02				
Tiller no.	0.49^***^	–0.09			
Leaf chlorophyll content	–0.06	0.27^*^	0.05		
Panicle no.	0.64^***^	–0.05	0.55^***^	–0.06	
Panicle length	–0.13	0.01	–0.26^*^	0.23^*^	–0.05

a Significance codes:

* 0.05 > P > 0.01;

** 0.01 > P > 0.001;

*** P < 0.001.

*Correlation Analysis for Weed-Competitive Ability Traits under Field Conditions in the 2014 DS*. Traits reported for weed-suppressive ability and weed tolerance are plant height, tiller number per plant, panicle number, flag-leaf length, flag-leaf width, leaf area, number of filled grains, number of unfilled grains, and grain yield per plant (Huel and Hucl 1996; Lanning et al. 1997; Lemerle et al. 1996). However, growth duration and flowering date were not associated with weed suppression (Zhao et al. 2006a); thus, these traits were not considered in the 2014 DS. Ability to produce yield and to suppress weeds is moderately heritable and closely associated under weed competition (Zhao et al. 2006a). Results of correlation analysis in this study showed that grain yield was positively and significantly correlated with all the traits and that all the traits were significantly and positively correlated with one another (P < 0.001) (Table 6). This also proves the claims of Caton et al. (2000) and Kruepl et al. (2007) that the complexity of traits for weed-competitive ability is controlled by the interaction of several traits rather than a single distinguishable trait.

The highest correlation (P < 0.001) with grain yield was recorded for filled grains (r = 0.99), followed by panicle number (r = 0.84) and percent spikelet fertility (r = 0.83) (Table 6), indicating a very strong correlation of these traits with yield, which is typically expected. Plant height and tiller number had the highest correlation with flag-leaf length (r = 0.83 and 0.81, respectively), followed by both flag-leaf width and leaf area (r = 0.82 and 0.78, respectively). Leaf area can be related to having a wider and longer flag leaf, which is essential for weed-competitive ability (Huel and Hucl 1996). Panicle number had the highest correlation with unfilled grains (r = 0.88) and filled grains (r = 0.86), whereas percent spikelet fertility had the highest correlation with flag-leaf length and flag-leaf width (r = 0.91). Percent spikelet fertility is a key trait for yield; thus, correlation analysis suggests that flag-leaf length and flag-leaf width are strong traits for yield in weed-competitive rice. Flag-leaf length and orientation could affect crop competitiveness because of their effects on light interception by the crop canopy (Huel and Hucl 1996).

**Table 6:** Correlation analysis of traits of the top 30 GSR IR2-6 introgression lines measured during the 2014 dry-season field experiment.^a^

Trait	Grain yield	Plant height	No. of tillers	No. of panicles	Flag-leaf length	Flag-leaf width	Leaf area	No. of filled grains	No. of unfilled grains
	—g plant–1—	—cm—			———cm———	—cm2—			
Grain yield (g plant–1)									
Plant height (cm)	0.62^***^								
No. of tillers	0.67^***^	0.81^***^							
No. of panicles	0 84^***^	0.62^***^	0.76^***^						
Flag-leaf length (cm)	0.76^***^	0.83^***^	0.81^***^	0.81^***^					
Flag-leaf width (cm)	0.77^***^	0.82^***^	0.78^***^	0.81^***^	0.98^***^				
Leaf area (cm2)	0.77^***^	0.82^***^	0.78^***^	0.76^***^	0.97^***^	0.98^***^			
No. of filled grains	0.99^***^	0.61^***^	0.67^***^	0.86^***^	0.76^***^	0.78^***^	0.77^***^		
No. of unfilled grains	0.64^***^	0.52^***^	0.65^***^	0.88^***^	0.68^***^	0.68^***^	0.64^***^	0.66^***^	
Percent spikelet fertility	0.83^***^	0.76^***^	0.71^***^	0.75^***^	0.91^***^	0.91^***^	0.89^***^	0.84^***^	0.48^***^

a Significance codes:

* 0.05 > P > 0.01;

** 0.01 > P > 0.001;

*** P < 0.001.

*Correlation Analysis for Weed-Competitive Ability Traits under Greenhouse Conditions*. Results of correlation analysis under greenhouse conditions showed that grain weight was positively and significantly correlated with all the traits and that all the traits were significantly and positively correlated with one another (P< 0.001), except for plant height at 7 DAS (P >0.05; r = 0.09) (unpublished data). The highest correlation with grain yield was recorded for maturity and yield-related traits: filled grains (r = 0.98) and panicle fresh weight (r = 0.77). The trait with the lowest correlation with grain weight was plant height at 7 DAS (r = 0.16). This implies that plant height at a very early stage (7 DAS) cannot be associated with the yield potential of ILs. Plant height at 7 DAS also has low correlation with panicle fresh weight and number of filled grains (r = 0.16), maximum plant height, flag-leaf length, and panicle number (r = 0.18).

*Correlation Analysis for Seedling Vigor Traits*. The traits associated with rapid seedling biomass accumulation are strongly associated with weed suppression and yielding ability under weed competition (Zhao et al. 2006b). Results of correlation analysis in Table 7 showed that vigor index was positively and significantly correlated with all the traits and that all the traits were significantly and positively correlated with one another, except for root dry weight and percent germination (P > 0.05; r = 0.07). All the rice traits were negatively correlated with weed density, weed fresh weight, and weed dry weight, except for root length, root fresh weight, and root dry weight. Leaf number at 28 DAS, tiller number, leaf dry weight, and total dry weight of ILs did not have any significant correlation with weed parameters. Likewise, leaf number at 7 DAS and leaf chlorophyll content (SPAD reading) were not associated with weed density. Few studies have been conducted on the contribution of root growth and activity to crop-weed competition. In rice, however, Fofana and Rauber (2000) found that competitive varieties tended to have desirable root growth. In this study, root length and root dry weight had no significant correlation with weed fresh weight (r = –0.01) and weed dry weight (r = 0.1), respectively.

**Table 7:** Correlation analysis of all traits for weed competitive ability in seedling vigor test.^a,b^

	%G	7PH	14PH	21PH	28PH	7LN	14LN	21LN	28LN	TN	RL	LCC	LFW	LDW	RFW	RDW	TFW	TDW	VI	WD	WFW
%G																					
7PH	0.63^***^																				
14PH	0.62^***^	0.80^***^																			
21PH	0.62^***^	0.84^***^	0.89^***^																		
28PH	0.38^***^	0.56^***^	0.63^***^	0.62^***^																	
7LN	0.54^***^	0.80^***^	0.73^***^	0.82^***^	0.46^***^																
14LN	0.61^***^	0.80^***^	0.86^***^	0.87^***^	0.58^***^	0.84^***^															
21LN	0.53^***^	0.75^***^	0.77^***^	0.85^***^	0.48^***^	0.84^***^	0.85^***^														
28LN	0.11^*^	0.32^***^	0.34^***^	0.32^***^	0.48^***^	0.46^***^	0.45^***^	0.56^***^													
TN	0.21^***^	0.42^***^	0.48^***^	0.44^***^	0.53^***^	0.52^***^	0.55^***^	0.64^***^	0.88^***^												
RL	0.18^***^	0.25^***^	0.26^***^	0.27^***^	0.36^***^	0.31^***^	0.36^***^	0.32^***^	0.40^***^	0.48^***^											
SPD	0.22^***^	0.29^***^	0.33^***^	0.33^***^	0.57^***^	0.27^***^	0.33^***^	0.25^***^	0.34^***^	0.43^***^	0.26^***^										
LFW	0.14^*^	0.39^***^	0.36^***^	0.31^***^	0.44^***^	0.35^***^	0.35^***^	0.39^***^	0.54^***^	0.56^***^	0.24^***^	0.29^***^									
LDW	0.16^**^	0.43^***^	0.44^***^	0.41^***^	0.60^***^	0.45^***^	0.46^***^	0.51^***^	0.72^***^	0.78^***^	0.31^***^	0.38^***^	0.64^***^								
RFW	0.17^**^	0.30^***^	0.25^***^	0.27^***^	0.22^***^	0.49^***^	0.42^***^	0.45^***^	0.60^***^	0.60^***^	0.47^***^	0.14^*^	0.34^***^	0.51^***^							
RDW	0.07	0.27^***^	0.19^***^	0.20^***^	0.21^***^	0.39^***^	0.32^***^	0.36^***^	0.56^***^	0.60^***^	0.45^***^	0.15^**^	0.30^***^	0.57^***^	0.77^***^						
TFW	0.16^**^	0.41^***^	0.37^***^	0.34^***^	0.44^***^	0.41^***^	0.40^***^	0.44^***^	0.60^***^	0.62^***^	0.30^***^	0.29^***^	0.99^***^	0.69^***^	0.49^***^	0.41^***^					
TDW	0.15^**^	0.43^***^	0.42^***^	0.39^***^	0.56^***^	0.47^***^	0.47^***^	0.52^***^	0.74^***^	0.80^***^	0.36^***^	0.36^***^	0.62^***^	0.98^***^	0.61^***^	0.71^***^	0.68^***^				
VI	0.88^***^	0.62^***^	0.63^***^	0.60^***^	0.42^***^	0.55^***^	0.62^***^	0.59^***^	0.32^***^	0.42^***^	0.21^***^	0.21^***^	0.34^***^	0.49^***^	0.38^***^	0.32^***^	0.38^***^	0.49^***^			
WD	−0.27^***^	−0.16^**^	−0.20^***^	−0.18^**^	−0.15^**^	−0.1	−0.12^*^	−0.17^**^	−0.01	−0.04	0.11	−0.07	−0.14^*^	−0.1	0.11	0.17^**^	−0.11^*^	−0.05	−0.28^***^		
WFW	−0.33^***^	−0.27^***^	−0.29^***^	−0.28^***^	−0.24^***^	−0.16^**^	−0.24^***^	−0.24^***^	−0.04	−0.07	−0.01	−0.12^*^	−0.16^**^	−0.1	0.05	0.12^*^	−0.14^**^	−0.06	−0.30^***^	0.66^***^	
WDW	−0.28^***^	−0.25^***^	−0.25^***^	−0.23^***^	−0.24^***^	−0.14^**^	−0.20^***^	−0.19^***^	−0.05	−0.09	0.1	−0.14^**^	−0.17^**^	−0.11	0.03	0.1	−0.15^**^	−0.07	−0.25^***^	0.56^***^	0.94^***^

a Abbreviations: %G, percent germination; 7PH, plant height at 7 d after sowing (DAS); 14PH, plant height at 14 DAS; 21PH, plant height at 21 DAS; 28PH, plant height at 28 DAS; 7LN, no. of leaves at 7 DAS; 14LN,no. of leaves at 14 DAS; 21LN, no. of leaves at 21 DAS; 28LN, no. of leaves at 21 DAS; LCC, leaf chlorophyll content; LFW, leaf fresh weight.; LDW, leaf dry weight; RFW, root fresh weight; RDW, root dry weight; RL,root length; TDW, total dry weight; TFW, total fresh weight; TN, tiller number; VI, vigor index; WD, weed density; WDW, weed dry weight; WFW, weed fresh weight.

b Significance codes:

^*^ 0.05≥P ≥0.01;

** 0.01≥P≥0.001;

*** P≤0.001.

*Correlation Analysis for Seed Germination Traits*. Results of correlation analysis in Table 8 showed that first-count germination (number of germinated seeds after 48 h) is positively and significantly correlated with second-count germination (number of germinated seeds after 7 d), percent germination, total fresh weight, total dry weight, and vigor index. However, first-count germination has a significant negative correlation with shoot and root length, average fresh weight, and average dry weight. Second-count germination and percent germination have a significant positive correlation with total fresh weight, total dry weight, and vigor index, but a nonsignificant negative correlation with shoot and root length.

**Table 8:** Correlation analysis for all the traits measured for weed competitive ability in seed germination test.^a^,^b^

	1st count	2nd count	% G	Root lt	Shoot lt	TFW	TDW	AFW	ADW
1st count									
2nd count	0.58^***^								
%G	0.57^***^	1.00^***^							
Root lt	−0.15^**^	−0.02	−0.02						
Shoot lt	−0.13^*^	−0.06	−0.06	0.37^***^					
TFW	0.30^***^	0.57^***^	0.57^***^	0.15^**^	0.11^*^				
TDW	0.49^***^	0.74^***^	0.73^***^	0.02	0.05	0.72^***^			
AFW	−0.40^***^	−0.66^***^	−0.63^***^	0.16^**^	0.13^*^	−0.04	−0.40^***^		
ADW	−0.21^***^	−0.45^***^	−0.41^***^	0.05	0.17^**^	0.04	0.11^*^	0.61^***^	
Vigor index^c^	0.58^***^	0.94^***^	0.94^***^	−0.02	−0.06	0.68^***^	0.86^***^	−0.51^***^	−0.26^***^

a Abbreviations: %G, percent germination; 1st count, first-count germination; 2nd count, second-count germination; AFW, average fresh weight; ADW, average dry weight; Root lt, root length; Shoot lt, shoot length; TFW, total fresh weight; TDW, total dry weight.

b Significance codes:

^*^ 0.05 > P > 0.01;

** 0.01 > P > 0.001;

*** P < 0.001.

c Vigor index was measured by computing percent germination x seedling dry weight.

Shoot and root length are significantly and positively correlated. These two traits have a nonsignificant positive correlation with total dry weight and nonsignificant negative correlation with vigor index. Finally, average dry weight is not correlated with root length and total fresh weight, whereas average fresh weight is significantly and negatively correlated with total dry weight. Diwan (2006) reported that first-count germination, rate of germination, and seedling dry weight were significantly correlated with vigor index. In this study, the same result was observed. Vigor index is strongly correlated with first-count germination, second-count germination, percent germination, total fresh weight, and total dry weight. However, vigor index has a strong negative correlation with average fresh weight and average dry weight, and a nonsignificant negative correlation with shoot length and root length.

*Regression Analysis for Weed-Competitive Ability Traits under Field Conditions in the 2013 WS*. Regression analysis was carried out to determine which traits were significantly affecting the grain yield of the newly developed introgression lines tested under weedy conditions in the 2013 WS. Using R version 1.5, the regression model was selected using all possible regression with leaps and the coefficient of variation (r2) as the criterion. The final regression model was YLD ~ Max.PH + Max.TN + SPD + PN + PL, which was significant at the 1% level (P < 0.001) with an r2 value of 0.4083 (Table 9).

**Table 9:** Test for significance of the regression model with coefficient of variation for yield.

Source	Degrees of freedom	F-computed	P-value	r2
Model^a^	78	12.46	6.821e-09	0.4083

a MODEL: YLD ∼ Max.PH + Max.TN + SPD + PN + PL.Abbreviations: Max.PH, maximum plant height (cm); Max.TN, maximum number of tillers; PL, panicle length (cm); PN, number of panicles; SPD, Soil-Plant Analysis (SPAD) reading.

This suggests that 40.83% of the variation in the yield of ILs can be attributed to the regression model. The variables with the highest r2 were tiller number and panicle number, indicating the importance of these variables in rice grain yield and weed-competitive ability of ILs in weedy environments. The fitted regression model showed that only number of panicles was significant at the 1% level using the standard t-test, indicating the importance of this trait to yield (Table 10). The regression coefficients for leaf chlorophyll content (SPAD reading) and panicle length were both negative. This implies that even very low values of these two traits can significantly increase the grain yield of the ILs. On the other hand, plant height, tiller number, and panicle number have positive regression coefficients, which means that these variables directly affect grain yield and are important traits under weed competition. These results coincide with the reports of Lemerle et al. (1996) and Lanning et al. (1997) that the characteristics that appear to confer competitiveness include crop height, tiller number, and percentage light interception.

**Table 10:** Regression coefficients of selected growth traits with grain yield as the response variable.

Trait	Regression coefficient	Standard error	t value	P-value^a^
(Intercept)	4.87553	6.24452	0.781	0.4373
Maximum plant height (cm)	0.01550	0.03918	0.396	0.6935
Maximum no. of tillers	0.35714	0.21367	1.671	0.0986
SPAD	-0.05280	0.11847	-0.446	0.6570
No. of panicles	1.28004	0.24787	5.164	1.8e-06 ^***^
Panicle length (cm)	-0.10548	0.19614	-0.538	0.5923

a Significance codes:

* 0.05 > P > 0.01;

** 0.01 > P > 0.001;

*** P < 0.001.

*Regression Analysis for Weed-Competitive Ability Traits under Field Conditions in the 2014 DS*. Regression analysis was performed to discover and identify the traits that were significantly affecting the grain yield of the ILs grown in the 2014 DS under weedy conditions. The final regression model was YLD ~ Max. PH + PN + FLL + FLW + LA+ Filled + Unfilled + SPF, which was significant at the 1% level (P < 0.001) with an r2 value of 0.9866 (Table 11). This signifies that 98.66% of the variation in the yield of the ILs can be attributed to the regression model. The variables with the highest r2 were panicle number, leaf area, filled grains, unfilled grains, and percent spikelet fertility, which might be an indication of the importance of these traits in the rice grain yield and weed-competitive ability of ILs in weedy environments. The fitted regression model shows that only the number of filled grains was significant at the 1% level using the standard t-test, indicating the significant contribution of this trait to grain yield (Table 12). The regression coefficients for the variables flag-leaf length, flag-leaf width, number of unfilled grains, and percent spikelet fertility are all negative, but only the number of unfilled grains was significant, which indicates that very low values of this variable can significantly affect the yield of the ILs. On the other hand, plant height, panicle number, leaf area, and number of filled grains are all positive, but only the number of filled grains is significant. This signifies that this variable is directly affecting grain yield and weed-competitive ability of the ILs.

**Table 11:** Test for significance of the regression model with coefficient of variation for yield.

Source	Degrees of freedom	F-computed	P-value	r2
Model^a^	80	809.8	< 2.2e-16	0.9866

a MODEL: YLD ~ Max.PH + PN + FLL + FLW + LA + Filled + Unfilled + SPF.Abbreviations: Filled, number of filled grains; FLL, flag-leaf length (cm); FLW, flag-leaf width (cm); LA, leaf area (cm2); Max.PH, maximum plant height (cm); PN, number of panicles; SPF, percent spikelet fertility; Unfilled, number of unfilled grains.

**Table 12:** Regression coefficients of selected growth traits with grain yield as the response variable.

Trait	Regression coefficient	Standard error	t value	P-value^a^
(Intercept)	–0.171495	0.127572	–1.344	0.1827
Maximum plant height (cm)	0.005995	0.003783	1.585	0.1170
No. of panicles	0.131534	0.082946	1.586	0.1167
Flag leaf length (cm)	–0.012463	0.052841	–0.236	0.8141
Flag leaf width (cm)	–1.392725	1.259102	–1.106	0.2720
Leaf area (cm2)	0.138974	0.083247	1.669	0.0989
No. of filled grains	0.023132	0.001081	21.399	< 2e-16^***^
No. of unfilled grains	–0.003142	0.001413	–2.224	0.0290^*^
Percent spikelet fertility	–0.012320	0.011722	–1.051	0.2964

a Significance codes:

* 0.05 ≥ P ≥ 0.01;

** 0.01 ≥ P ≥ 0.001;

*** P ≤ 0.001.

**Phenotypic Variations in Traits for Weed-Competitive Ability under Field Conditions**. To successfully improve a trait through breeding, variation must exist in the trait among the available germplasm and the trait must be heritable. A significant portion of the observable phenotypic variation expressed among genotypes must be attributed to genotypic differences (Fehr et al. 1987). There is high variation in all the phenotypic traits observed, which implies high genotypic variation for the investigated traits among the ILs. The traits that showed the highest coefficient of variation (CV) values were number of unfilled grains (79.6%), grain yield per plant (79.3%), number of filled grains (78.2%), panicle number (65.2%), and percent spikelet fertility (56.3%). The traits with CV values lower than 50% were plant height (34.9%), tiller number (43.2%), flag-leaf length (46.3%), flag-leaf width (48.7%), and leaf area (49.8%). The mean grain yield per plant in the 2014 DS was 4.03 g (SD = 3.19), with an average of four tillers and three panicles. The yield performance of each IL varied with weed regime. To verify this observation, the ILs were analyzed for the significance of genotypic effect per weedy condition, specifically to determine whether each genotype has a differential yield response because of varying weed conditions. The fixed-effects model was used for the ANOVA with the Satterthwaite denominator. The summary of the ANOVA (Table 13) shows a significant genotypic effect on UPW (F = 2.47, P = 0.0095), UPWF (F = 2.74, P = 0.0000), and LLW (F = 1.78, P = 0.0000) treatments at the 1% level of significance. The summary of ANOVA (Table 14) shows significant genotypic effects on plant height, flag-leaf length, flag-leaf width, and leaf area for all weed regimes. Yield-related traits such as number of tillers, number of panicles, number of filled and unfilled grains, and grain yield were significantly affected by genotype only for UPWF and UPW conditions. In LLW conditions, no significant genotypic effect was found on yield-related traits: number of tillers, number of panicles, number of filled and unfilled grains, and grain yield. This suggests that selection for the weed-competitive genotypes was stronger for upland conditions than for lowland conditions.

**Table 13:** ANOVA for testing the significance of genotype effect in upland weedy, upland weed-free, and lowland weedy conditions in the 2014 dry season.

Condition	Sum of squares	Mean square	F value	Satterthwaite denominator	P-value
Upland weedy	239,508.0	1,228.25	2.47	20.51	0.0095^**^
Upland weed-free	345,514.8	1,771.87	2.74	382.99	0.0000^***^
Lowland weedy	302,536.4	1,551.47	1.78	194.99	0.0000^***^

a Significance codes:

* 0.05 > P > 0.01;

** 0.01 > P > 0.001;

*** *** P < 0.001.

**Table 14:** ANOVA for testing the significance of genotype effect per trait.^a^

Trait	Condition^b^	Sum of squares	Mean square	F value	Satterthwaite denominator	P-value
Plant height (cm)	LLW	302,536.4	1,551.5	1.8	195.0	0.0000^***^
	UPWF	345,514.8	1,771.9	2.7	383.0	0.0000^***^
	UPW	239,508.0	1,228.2	2.5	20.5	0.0095^**^
No. of tillers	LLW	3,354.1	17.2	17.2	40.9	0.1366
	UPWF	3,537.8	18.1	2.2	195.0	0.0000^***^
	UPW	2,446.1	12.5	2.4	195.0	0.0000^***^
No. of panicles	LLW	581.1	3.0	1.1	22.5	0.3808
	UPWF	3,163.3	16.2	2.2	27.3	0.0089^**^
	UPW	3,187.5	16.3	2.1	196.0	0.0000^***^
Flag-leaf length (cm)	LLW	20,129.6	103.2	1.6	383.9	0.0000^***^
	UPWF	21,498.5	110.2	2.2	371.6	0.0000^***^
	UPW	15,973.4	81.9	2.0	28.4	0.0182^*^
Flag-leaf width (cm)	LLW	43.8	0.2	1.7	383.7	0.0000^***^
	UPWF	55.2	0.3	1.9	55.4	0.0022^**^
	UPW	42.0	0.2	2.1	38.3	0.0045^**^
Leaf area (cm2)	LLW	10,791.6	55.3	1.7	383.9	0.0000^***^
	UPWF	12,381.9	63.5	1.9	69.8	0.0017^**^
	UPW	8,417.7	43.2	2.1	31.4	0.0082^**^
Grain yield (g plant–1)	LLW	544.4	2.8	1.2	7.2	0.4223
	UPWF	9,764.7	50.1	2.1	383.8	0.0000^***^
	UPW	3,492.8	17.9	1.7	377.3	0.0000^***^
No. of filled grains	LLW	910,740	4670	1.2	10.2	0.3905
	UPWF	18,028,276	92,453	2.2	383.6	0.0000^***^
	UPW	7,219,466	37,023	1.7	379.0	0.0000^***^
No. of unfilled grains	LLW	1,329,957	6,820	1.1	21.9	0.4112
	UPWF	7,070,223	36,258	2.0	383.8	0.0000^***^
	UPW	7,632,774	39,142	2.1	196.0	0.0000^***^

a Significance codes:

* 0.05 ≥ P ≥ 0.01;

** 0.01 ≥ P ≥ 0.001;

*** P < 0.001.

b Abbreviations: LLW, lowland weedy; UPW, upland weedy; UPWF, upland weed-free.

**Phenotypic Variations in Traits for Weed-Competitive Ability under Greenhouse Conditions**. High phenotypic variations were observed under greenhouse conditions, indicating high genotypic variation for the investigated traits among the ILs. The highest CV values were observed for panicle fresh weight (77.1%), number of unfilled grains (72.8%), grain yield per plant (68.9%), number of filled grains (67.7%), and percent germination (52.2%). Traits with CV values lower than 50% were plant height at 21 DAS (21.7%), maximum plant height (23.7%), plant height at 14 DAS (26.3%), flag-leaf length (29.4%), and flag-leaf width (33.0%). The summary of ANOVA (unpublished data) shows significant genotypic effects on all traits measured in the greenhouse experiment at the 1% level of significance. The genotypes (ILs) differed significantly in terms of germination, seedling vigor, canopy traits, and maturity and yield-related traits.

**Phenotypic Variations in Seedling Vigor Traits**. High variation in all the phenotypic traits was observed in the seedling vigor experiment. This implies high genotypic variation for the investigated traits between the ILs. Traits that showed the highest CV values were vigor index (50.1%), initial germination and percent germination (45.4%), root fresh weight (45.3%), root dry weight (40.5%), and leaf fresh weight (39.7%). Traits with CV values lower than 30% were plant height at 28 DAS (15.0%), number of tillers (22.3%), SPAD reading (23.3%), and number of leaves at 28 DAS (25.8%).

The summary of ANOVA (Table 15) shows significant genotypic effects on all traits measured (P < 0.0001) at the 1% level of significance, except for plant height at 7 DAS (P = 0.174). Seedling vigor traits with highly significant variations include plant height at 28 DAS, leaf number at 28 DAS, number of tillers, root fresh weight, and root dry weight. Plant height at 21 DAS was significant only at the 5% level.

**Table 15:** ANOVA for testing the significance of genotype effect per seedling vigor trait.^a^

Trait^b^	Sum of squares	Mean square	*F* value	*P*-value
Percent germination	218,771.26	1,317.90	1.44	0.0093^**^
Plant height at 7 DAS (cm)	6,096.06	36.72	1.16	0.1742
Plant height at 14 DAS (cm)	17,092.21	102.97	1.49	0.0050^**^
Plant height at 21 DAS (cm)	39,066.48	235.34	1.31	0.0409^*^
Plant height at 28 DAS (cm)	38,934.83	234.55	2.19	0.0000^***^
No. of leaves at 7 DAS	130.79	0.79	1.64	0.0008^***^
No. of leaves at 14 DAS	379.61	2.29	1.62	0.0010^***^
No. of leaves at 21 DAS	2,206.21	13.29	1.71	0.0003^***^
No. of leaves at 28 DAS	4,559.49	27.47	2.10	0.0000^***^
No. of tillers	259.26	1.56	2.64	0.0000^***^
Leaf chlorophyll content	17,313.03	104.30	1.48	0.0061^**^
Root length (cm)	5,372.42	32.36	1.67	0.0005^***^
Leaf fresh weight (g plant^-1^)	1,801.33	10.85	1.49	0.0055^**^
Leaf dry weight (g plant^-1^)	21.26	0.13	1.56	0.0023^**^
Root fresh weight (g plant^-1^)	63.38	0.39	2.24	0.0000^***^
Root dry weight (g plant^-1^)	1.29	0.01	1.97	0.0000^***^
Total fresh weight (g plant^-1^)	2,213.23	13.33	1.61	0.0012^**^
Total dry weight (g plant^-1^)	30.12	0.18	1.66	0.0006^***^
Vigor index	375,273.85	2,260.69	1.56	0.0023^**^

a Significance codes:

* 0.05 > P > 0.01;

** 0.01 > P > 0.001;

*** P < 0.001.

b Abbreviation: DAS, days after sowing.

**Phenotypic Variation in Seed Germination Traits**. High phenotypic variation for early germination was observed in the seed germination test, indicating high genotypic variation within the ILs. Traits that showed the highest CV values were relative fresh weight (48.3%), first-count germination (44.1%), relative dry weight (33.3%), and vigor index (31.5%). Traits with CV values lower than 25% were total dry weight (17.8%), shoot length (18.6%), root length (24.5%), total fresh weight (17.8%), and second-count germination (21.9%). The summary of ANOVA shows significant genotypic effects on all traits measured (P < 0.0001) at the 1% level, except for root length (P = 0.0834) (Table 16). Shoot length was significant only at the 5% level. This implies that the ILs had comparable shoot and root lengths without any distinct differences. Seed germination and vigor traits that showed highly significant variations include first-count germination, second-count germination, percent germination, total fresh weight, total dry weight, and vigor index. Diwan (2006) reported similar results in a doubled-haploid population of IR64/‘Azucena’.

**Table 16:** ANOVA for testing the significance of genotype effect per seed germination trait.^a^

Trait	Sum of squares	Mean square	F value	P-value
First-count germination	8,382.95	50.50	2.88	0.0000^***^
Second-count germination	7,918.75	47.70	3.88	0.0000^***^
Percent germination	128,918.71	776.62	3.79	0.0000^***^
Shoot length (cm)	33,068.21	199.21	1.41	0.0137^*^
Root length (cm)	54,759.69	329.88	1.24	0.0834
Total fresh weight (g)	8.19	0.05	2.96	0.0000^***^
Total dry weight (g)	1.79	0.0107	4.06	0.0000^***^
Average fresh weight (g)	0.12	0.0007	1.44	0.0099^**^
Average dry weight (g)	0.01	0.0001	1.83	0.0001^***^
Vigor index	23,039.10	138.79	3.59	0.0000^***^

a Significance codes:

* 0.05 ≥ P ≥ 0.01;

** 0.01 ≥ P ≥ 0.001;

*** P ≤ 0.001.

**Figure 5:**
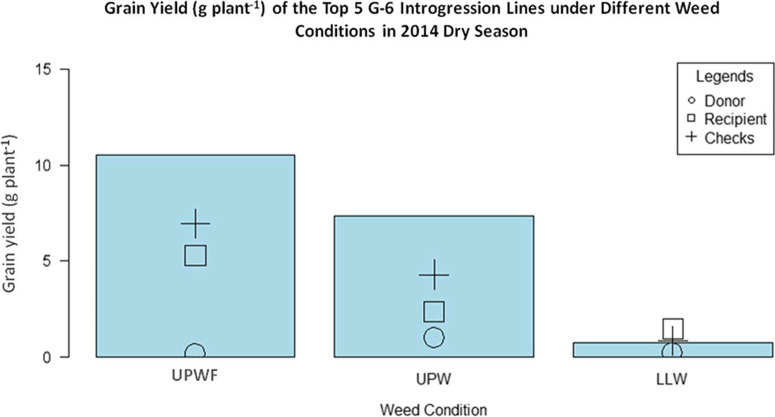
Grain yield (g plant–1) of the top 5 GSR IR2-6 (G-6) introgression lines (ILs) under upland weedy (UPW), upland weed-free (UPWF), and lowland weedy (LLW) conditions in the 2014 dry season. Bars represent mean grain yield (g plant–1) of the G-6 ILs.

**Promising ILs with Superior Traits and Weed-Competitive Ability**.Figure 5 shows that, under UPWF and UPW conditions, the top five ILs had higher grain yields than the recurrent parent (WTR1), donor parent (Y134), and checks (PSB Rc82 and Apo). This suggests that these ILs have strong weed-competitive ability and are able to produce comparable yields despite the presence of competing weeds. In LLW conditions, the ILs did not perform very well, having very low grain yield. This implies that selection for weed-competitive rice genotypes is stronger in upland conditions than in lowland conditions. Recipient parent WTR1 had the highest grain yield under LLW conditions, while the donor parent Y134 had the lowest grain yield across all weedy conditions. However, the grain yield of the checks was higher than that of both parents under UPWF and UPW conditions.

Table 17 shows the grain yield of the top five ILs in UPWF, UPW, LLW, and greenhouse conditions and their corresponding yield difference with WTR1 (recipient parent), Y134 (donor parent), and checks PSB Rc82 and Apo. The ILs had the highest grain yield across weed regimes. Check cultivar Apo had higher grain yield than the parents (WTR1 and Y134). G-6-Y9-WU-2 (21.13 g plant–1), G-6-Y7-WL3 (12.43 g plant–1), and G-6-L2-WL-3 (15.28 g plant–1) were the highest-yielding ILs under UPWF, UPW, and LLW conditions, respectively (Table 17).

**Table 17:** Grain yield (g plant-1) of top 5 performing GSR IR2-6 introgression lines and their yield difference with parents (WTR1 and Y134) and checks (PSB Rc82 and Apo) under upland weed-free, upland weedy, lowland weedy, and greenhouse conditions in the 2014 dry season.

Condition	Rank (top 1–5)	Introgression line	Grain yield	WTR 1	Y134	Yield difference^a^	Apo	Mean
						PSB Rc82		
Upland weed-free					g plant–1			
						
				5.27	0.2	4.17	7.43	
	1	G-6-Y9-WU2	21.13	15.86*	20.93*	16.96*	13.7	16.86
	2	G-6-Y5-WU3	20.11	14.84	19.91*	15.94*	12.68	15.84
	3	G-6-L6-WU1	20.07	14.8	19.87*	15.90*	12.64	15.8
	4	G-6-L5-WU1	19.27	14	19.07*	15.1	11.84	15
	5	G-6-Y10-WU3	18.44	13.17	18.24*	14.27	11.01	14.17
				2.36	3.76	1.1	5.29	
Upland weedy	1	G-6-Y7-WL3	12.43	10.07*	8.67*	11.33*	7.14	9.3
	2	G-6-Y7-WL2	12.17	9.81*	8.41*	11.07*	6.88	9.04
	3	G-6-Y6-WU3	11.96	9.60*	8.20*	10.86*	6.67	8.83
	4	G-6-Y3-WL3	11.59	9.23*	7.83*	10.49*	6.3	8.46
	5	G-6-Y8-WU1	10.77	8.41	7.01	9.67	5.48	7.64
				1.92	0.75	1.19	1.09	
Lowland weedy	1	G-6-L2-WL3	15.28	13.36*	14.53*	14.09*	14.19*	14.04
	2	G-6-RF6-WL3	7.35	5.43*	6.60*	6.16*	6.26*	6.11
	3	G-6-L15-WU1	6.21	4.29*	5.46*	5.02*	5.12*	4.97
	4	G-6-RF7-WL3	5.49	3.57	4.74	4.3	4.4	4.25
	5	G-6-Y16-WL2	4.5	2.58	3.75	3.31	3.41	3.26
				5.25	3.41	11.9	12.3	
Greenhouse	1	G-6-L9-WU2	18.27	13.02	14.86*	6.37	5.97	10.06
	2	G-6-RF13-WU1	16.02	10.77	12.61*	4.12	3.72	7.81
	3	G-6-L5-WU2	15.21	9.96	11.80*	3.31	2.91	7
	4	G-6-L6-WU1	14.3	9.05	10.89*	2.4	2	6.09
5	G-6-RF12-WU1	12.39	7.14	8.98	0.49	0.09	4.18

a Italics signify mean or average value. Asterisks denote significant at the 5% level.

In UPWF conditions, only G-6-Y9-WU-2 had a significant yield difference with WTR1, whereas the yield differences between the top five ILs and Y134 were significant at the 0.5% level. G-6-Y9-WU-2, G-6-Y5-WU-3, and G-6-L6-WU-1 had significant yield differences with PSB Rc82. The yield differences between the top five ILs and check cultivar Apo were not significant.

In UPW conditions, G-6-Y7-WL3, G-6-Y7-WL2, G-6-Y6-WU-3, and G-6-Y3-WL3 had significant yield differences with WTR1, Y134, and PSB Rc82. The yield differences between the top five ILs and check cultivar Apo were not significant.

In LLW conditions, G-6-L2-WL-3, G-6-R6-WL3, G-6-L15-WU1, and G-6-L15-WU-1 had significant yield differences with all the parents and checks. In greenhouse conditions, only Y134 had a significant yield difference with the top four ILs: G-6-L9-WU-2, G-6-RF13-WU-1, G-6-L5-WU-2, and G-6-L6-WU-1.

The maximum plant height of ILs, parents, and checks did not differ across weed regimes. However, G-6-Y9-WU-2, G-6-Y7-WL-3, G-6-RF7-WL-3, and G-6-RF13-WU1 were the tallest in UPWF, UPW, LLW, and greenhouse conditions, respectively.

Leaf area is an important trait for weed competitiveness, as it affects competition for light. Flag-leaf length and width are two important traits for determining leaf area. G-6-L6-WU-1, G-6-Y6-WU-3, G-6-L2-WL-3, and G-6-L5-WU-2 had the largest leaf area under UPWF, UPW, LLW, and greenhouse conditions, respectively.

Compared with the parents and checks, the ILs had a higher number of filled grains and percent spikelet fertility and a lower number of unfilled grains. These traits are important components in attaining high grain yield. This suggests that, despite the presence of weed competition, these top five ILs were the most promising for producing higher yields and better morphological and agronomic traits in each condition.

**Promising ILs for Replicated Yield Trials**. Across the three conditions (UPWF, UPW, and LLW), 21 lines were selected as the most promising ILs based on yield ability and percent spikelet fertility (Table 18). Among the ILs, G-6-Y5-WU-3, G-6-L6-WU-1, and G-6-Y10-WU-3 were the three highest-yielding lines in UPWF conditions. G-6-L2-WL-3, G-6-RF6-WL-3, G-6-L15-WU-1, G-6-Y16-WL-2, and G-6-L6-WU-3 were the top ILs in LLW conditions, whereas G-6-Y7-WL-3, G-6-Y6-WU-3, G-6-Y3-WL-3, and G-6-Y8-WU-1 were the highest yielding in UPW conditions. In greenhouse conditions, G-6-L9-WU-2, G-6-RF13-WU-1, G-6-L5-WU-2, G-6-L6-WU-1, and G-6-RF12-WU-1 were the most promising.

**Table 18:** Twenty-one most-promising GSR IR2-6 introgression lines from upland weed-free (UPWF), upland weedy (UPW), lowland weedy (LLW), and greenhouse (SHW) conditions in the 2014 dry season.^a^

Rank (1–21)	Condition	Introgression line		Grain yield	Percent spikelet fertility	
			—g plant–1—			
1	UPWF	G-6-Y5-WU3		20.11	86.04	
2	UPWF	G-6-L6-WU1		20.07	77.50	
3	UPWF	G-6-Y10-WU3		18.44	73.68	
4	SHW	G-6-L9-WU2		18.27	66.01	
5	UPWF	G-6-Y13-WU2		18.07	76.61	
6	UPWF	G-6-Y8-WU1		17.38	68.33	
7	UPWF	G-6-Y12-WU3		16.68	79.56	
8	SHW	G-6-RF13-WU1		16.02	71.23	
9	LLW	G-6-L2-WL3		15.28	88.73	
10	SHW	G-6-L5-WU2		15.21	71.48	
11	SHW	G-6-L6-WU1		14.30	93.65	
12	UPW	G-6-Y7-WL3		12.43	72.05	
13	SHW	G-6-RF12-WU1		12.39	68.31	
14	UPW	G-6-Y6-WU3		11.96	66.11	
15	UPW	G-6-Y3-WL3		11.59	63.28	
16	UPW	G-6-Y8-WU1		10.77	68.01	
17	UPW	G-6-Y9-WU1		9.56	77.51	
18	LLW	G-6-RF6-WL3		7.35	66.84	
19	LLW	G-6-L15-WU1		6.21	81.67	
20	LLW	G-6-Y16-WL2		4.50	76.39	
21	LLW	G-6-L6-WU3		4.23	89.07	

a Selected based on grain yield and percent spikelet fertility.

In summary, correlation analysis on G-6 ILs grown under weedy conditions showed that grain yield was positively and significantly correlated with all the traits and that all the traits were significantly and positively correlated with one another. This confirms previous reports that the complexity of traits for weed-competitive ability is controlled by the interaction of several traits rather than a single desirable trait. Regression analysis indicated that panicle number, leaf area, number of filled grains, number of unfilled grains, and percent spikelet fertility are the important traits for grain yield of G-6 ILs under weed competition. Selection for weed-competitive genotypes was stronger in upland conditions than in lowland conditions. Significant genotypic effects were observed for plant height, flag-leaf length, flag-leaf width, and leaf area for all weed regimes. However, yield-related traits such as number of tillers, number of panicles, number of filled and unfilled grains, and grain yield were significantly affected by genotype only for UPWF and UPW conditions.

In weedy conditions, the mean yield of the G-6 ILs was significantly higher than the mean yield of WTR1, Y134, and checks PSB Rc82 and Apo. The yield performance of ILs was directly affected by traits for weed-competitive ability; thus, G-6 ILs are better weed competitors and can produce comparable yields despite the presence of weeds.

The height of plants at a very early stage (7 DAS) cannot be clearly associated with the yield ability of ILs and other important agronomic traits. A seedling vigor test showed that all traits are significantly and positively correlated with one another, except for root dry weight and percent germination. All the traits are negatively correlated with weed density, weed fresh weight, and weed dry weight, except for root length, root fresh weight, and root dry weight.

A germination test of G-6 ILs indicated that vigor index is significantly and strongly associated with first-count germination, second-count germination, percent germination, total fresh weight, and total dry weight. However, vigor index had a significant and strong negative correlation with relative fresh and dry weights and a nonsignificant correlation with shoot and root lengths.

After three cycles of selection process across weedy conditions, 21 most-promising introgression fixed lines showing superior traits and weed-competitive ability were identified. The successful development of weed-competitive rice cultivars will provide an effective new strategy to manage weeds with a single hand weeding or herbicide application. This will result in lower labor costs, less herbicide use, prevention of possible development of herbicide-resistant weeds, and increased profit for rice farmers. Furthermore, the use of weed-competitive rice varieties will significantly improve yields in aerobic rice systems and can become an important strategy that complements water-saving techniques for promoting the adoption of upland rice production.
